# Metabolic Obesity Phenotypes and Risk of Cellulitis: A Cohort Study

**DOI:** 10.3390/jcm8070953

**Published:** 2019-06-30

**Authors:** Hae Suk Cheong, Yoosoo Chang, Eun-Jung Joo, Ara Cho, Seungho Ryu

**Affiliations:** 1 Division of Infectious Disease, Department of Internal Medicine, Kangbuk Samsung Hospital, Sungkyunkwan University School of Medicine, Seoul 03181, Korea; 2 Center for Cohort Studies, Total Healthcare Center, Kangbuk Samsung Hospital, Sungkyunkwan University, School of Medicine, Seoul 04514, Korea; 3 Department of Occupational and Environmental Medicine, Kangbuk Samsung Hospital, Sungkyunkwan University School of Medicine, Seoul 03181, Korea; 4 Department of Clinical Research Design & Evaluation, SAIHST, Sungkyunkwan University, Seoul 06351, Korea; 5 Department of Epidemiology and Medical Informatics, School of Public Health, Korea University, Seoul 02841, Korea

**Keywords:** cellulitis, obesity, cohort study, metabolically healthy obesity

## Abstract

No cohort studies have evaluated the effect of obesity on the risk of cellulitis according to metabolic health status. We investigated an association of BMI and metabolic health status with the development of cellulitis. We conducted a cohort study of 171,322 Korean adults who underwent a health checkup examination and were followed from 2011 to 2016 for cellulitis and hospital admission related to cellulitis, which were ascertained through the linkage to the Health Insurance and Review Agency database. Being metabolically healthy was defined as not having any metabolic syndrome component and having a homeostasis model assessment of insulin resistance <2.5. During 638,240.4 person-years of follow-up, 14,672 cases of incident cellulitis were identified with 225 cases of cellulitis-related admission. After adjustment for possible confounders, the multivariable-adjusted hazard ratios (95% CI) for incident cellulitis comparing BMIs 23–24.9, 25–29.9, and ≥30 with a BMI of 18.5–22.9 kg/m^2^ as the reference were 1.07 (1.02–1.11), 1.09 (1.04-1.13), and 1.19 (1.08–1.31), respectively, whereas the corresponding multivariable-adjusted hazard ratios (95% CI) for cellulitis-related admission were 1.55 (1.05–2.3), 2.47 (1.73–3.53), and 4.8 (2.86–8.05), respectively. These associations were consistently observed in both metabolically healthy and unhealthy individuals with no significant interaction. In a large cohort of apparently healthy adults, increased BMI was associated with an increased risk of cellulitis and hospitalization for cellulitis in both metabolically healthy and unhealthy individuals. Obesity appears to be an independent risk factor for cellulitis regardless of metabolic phenotype.

## 1. Introduction

Cellulitis is a bacterial skin and soft tissue infection, characterized by erythema, swelling, warmth, and pain that occurs when the physical skin barrier, the immune system, and/or the circulatory system are impaired [1,2,3]. Cellulitis is a common global burden and one of the most common bacterial infections requiring hospitalization [4,5].

Obesity is becoming increasingly prevalent worldwide and is associated with a number of chronic diseases including cardiovascular disease, type 2 diabetes mellitus, and certain cancers [6,7]. Furthermore, previous studies have suggested that obesity is more likely to increase the risk of various types of infections [8]. Obesity has been reported to be positively associated with occurrence, recurrence, treatment failure, and longer treatment of cellulitis in retrospective chart review studies or case–control studies in hospital-based settings [9,10,11,12,13]. Until now, only a cohort study of Danish blood donors showed that obesity increased the risk of skin and subcutaneous tissue infection only in men, but this study was limited by a small number of skin and soft tissue infection cases and self-reported anthropometric data [14]. Moreover, prevalence of obesity-related metabolic abnormalities varies among obese individuals showing different obesity metabolic phenotypes. A proportion of obese subjects, referred to as metabolically healthy obese, appear to have a favorable metabolic profile with no metabolic abnormalities and has been reported to have favorable prognosis [15,16]. Currently, it is yet unclear whether the relationship between obesity and cellulitis is attributed to obesity, per se, or the presence of coexisting metabolic abnormalities. So far, there have been no studies on the relationship between obesity and cellulitis according to metabolic health status.

Therefore, we performed a longitudinal cohort study to evaluate the association of body mass index (BMI) with the risk of cellulitis and its related hospital admission in metabolically healthy and unhealthy adults free of cellulitis at baseline. Participants underwent a health screening examination program while using a strict criterion with zero metabolic abnormalities and with no insulin resistance to define the metabolically healthy phenotype, as previously applied [17,18].

## 2. Methods

### 2.1. Study Population

The Kangbuk Samsung Health Study (KSHS) is a cohort study of Korean men and women aged 18 years and over who underwent a comprehensive annual or biennial health examination at the Kangbuk Samsung Hospital Total Healthcare Centers in Seoul and Suwon, South Korea [17]. Most examinees (over 80%) and their spouses are employees of various companies and local governmental organizations. In South Korea, Industrial Safety and Health Law requires annual or biennial health screening examinations of all employees, free of charge. The rest of the examinees voluntarily purchased health screening exams at the healthcare center.

**Figure 1:**
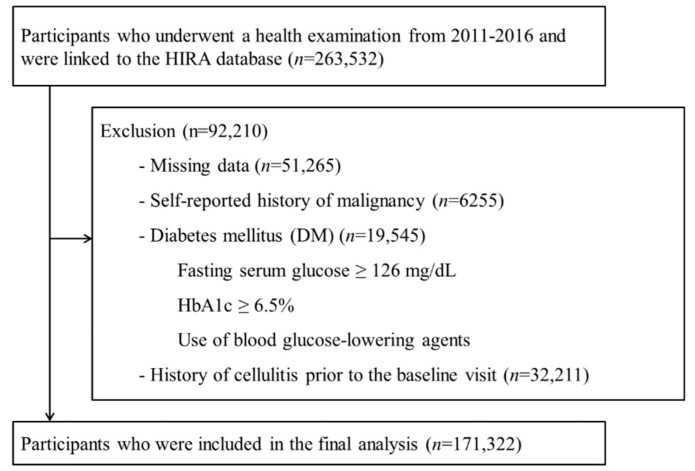
Selection of the study population. HIRA = Health Insurance Review and Assessment Service.

This cohort study involved a portion of the Kangbuk Samsung Health Study participants who underwent a comprehensive health exam from 2011 to 2016 and gave informed consent for linkage to the Health Insurance Review and Assessment Service (HIRA) database (*n* = 263,532). Among them, 92,210 participants were excluded at baseline due to missing data on BMI, glucose, blood pressure (BP), high-density lipoprotein cholesterol (HDL-C), triglycerides, homeostasis model assessment of insulin resistance (HOMA-IR) and high-sensitivity C-reactive protein (hsCRP) (*n* = 51,265), a history of malignancy (*n* = 6255), diabetes mellitus including a fasting serum glucose ≥126 mg/dL, HBA1c ≥ 6.5%, use of blood glucose-lowering agents (*n* = 19,545), or a diagnosis of cellulitis prior to the baseline visit (*n* = 32,211). Because some participants met more than one exclusion criterion, the total number of participants included in the final analysis was 171,322 (Figure 1). Written informed consent was obtained from all participants, and the study was approved by the Institutional Review Board of the Kangbuk Samsung Hospital.

### 2.2. Measurements

Data on demographic characteristics, lifestyle factors, education level, and medical history of cardiovascular disease or cancer were collected by standardized, self-administered questionnaires at the health screening visit [17]. Education level was categorized as less than college graduate vs. college graduate or more. The questionnaire asked about the frequency of alcohol drinking and amount of alcohol consumed per drinking day, recorded in standard units [19]. Average alcohol consumption per day was calculated using the frequency and number of beverages consumed per drinking day. Alcohol consumption was categorized into none, moderate (≤20 g/d), and high (>20 g/d). Smoking status was categorized as never, former, or current smoker. Physical activity was assessed using the validated Korean version of the International Physical Activity Questionnaire (IPAQ) short form [20]. Participants were classified into inactive, minimally active, or health-enhancing physical activity (HEPA) [21]. HEPA was defined as physical activity that met either of two criteria: (i) vigorous intensity activity on three or more days per week accumulating ≥1500 metabolic equivalent (MET) min/week; or (ii) seven days of any combination of walking, moderate intensity, or vigorous intensity activities achieving at least 3000 MET min/week [20]. Typical dietary consumption was assessed using a 106-item, self-administered food frequency questionnaire (FFQ) designed and validated for use in Korea [22].

Blood pressure, height, and weight were measured by trained nurses. Height was measured to the nearest 0.1 cm using a stadiometer with the examinee standing without shoes. Weight was measured to the nearest 0.1 kg in a light gown while barefoot using a bioimpedance analyzer (Inbody 720, Biospace Co., Seoul, Korea), which was calibrated every day before beginning the tests. BMI was categorized according to Asian-specific criteria [23]: underweight, BMI <18.5 kg/m^2^; normal weight, BMI of 18.5 to 22.9 kg/m^2^; overweight, BMI of 23 to 24.9 kg/m^2^; obese I, BMI of 25 to 29.9 kg/m^2^, and obese II, BMI ≥30 kg/m^2^. Metabolically unhealthy persons were defined as those having at least one of the following metabolic abnormalities [18]: (1) fasting glucose level ≥100 mg/dL; (2) BP ≥130/85 mmHg or current use of BP-lowering agents; (3) elevated triglyceride level (≥150 mg/dL) or current use of lipid-lowering agents; (4) low HDL-C (<40 mg/dL in men or <50 mg/dL in women); or (5) insulin resistance, defined as HOMA-IR score ≥2.5 [24]. Otherwise, being metabolically healthy was defined as none of the metabolic abnormalities described above, as previously applied [17,18].

Blood specimens were sampled from the antecubital vein after at least 10 h of fasting. The blood tests included total cholesterol, low-density lipoprotein cholesterol (LDL-C), HDL-C, triglycerides, alanine transaminase (ALT), fasting glucose, insulin, uric acid, and high-sensitivity C-reactive protein (hsCRP). The homeostasis model assessment of insulin resistance (HOMA-IR) was calculated as fasting insulin (mg/dL)×fasting glucose (mg/dL)/405 [24]. Hypertension was defined as a systolic BP ≥140 mmHg, a diastolic BP ≥90 mmHg, or the use of antihypertensive medications. Diabetes mellitus was defined as a fasting serum glucose ≥126 mg/dL, HBA1c ≥ 6.5%, or use of blood glucose-lowering agents.

### 2.3. Ascertainment of Cellulitis, Cellulitis-Related Hospitalization, and Comorbidity Index

In Korea, health care is organized under a mandatory single-payer nationwide insurance system (National Health Insurance, NHI) that collects all information on medical services utilization covering the entire Korean population under a comprehensive database operated by HIRA [25]. Primary outcome was defined as a diagnosis of cellulitis (ICD-10, L03 [6]) at either outpatient or hospitalization ascertained through linkage to the HIRA database by the end of 2016. We also separately evaluated incident cellulitis-related hospitalization, defined as the first hospital admission with a principal or secondary diagnosis of cellulitis [6].

We used Charlson’s Comorbidity Index to adjust comorbidity as a potential confounding factor. This score includes 17 major disease categories identified by ICD-10 codes and includes congestive heart failure, chronic pulmonary disease, cerebrovascular disease, dementia, rheumatic disease, diabetes, renal disease, cancer, liver disease, and acquired immune deficiency syndrome [26].

### 2.4. Statistical Analyses

The characteristics of the study participants were explored according to the BMI categories (<18.5, 18.5–22.9, 23–24.9, 25–29.9, or ≥30 kg/m^2^). To compare results among different BMI categories, one-way analysis of variance (ANOVA) was used for continuous variables, and a chi-square test was used for categorical variables.

The primary endpoint was development of cellulitis and cellulitis-related hospitalization. Each participant was followed from the baseline exam until either development of incident cellulitis or the end of 2016, whichever came first. Hazard ratios (HRs) and 95% confidence intervals (CIs) for incident cellulitis were estimated using Cox proportional hazards regression analysis. We assessed the proportional hazards assumption by examining graphs of estimated log (−log(survival)); no violation of the assumption was found.

The models were initially adjusted for age and sex and then were further adjusted for potential confounders, including study center (Seoul or Suwon), year of screening exam (one-year categories), smoking, alcohol intake, physical activity, education level, total energy intake, and Charlson’s comorbidity index (Model 1). Model 2 was further adjusted for LDL cholesterol, HDL cholesterol, triglycerides, glucose, systolic blood pressure, hs-CRP, and HOMA-IR (Model 2). To test for linear trends, the number of categories were used as continuous variables in each regression model.

All statistical analyses were conducted with Statistical Analysis Software (SAS) Enterprise Guide Version 6.1 (SAS Institute, Inc., Cary, NC, USA). All *p* values less than 0.05 were considered statistically significant.

## 3. Results

The characteristics of the cohort are presented in Table 1. The mean age (standard deviation) and mean BMI (SD) of 171,322 participants were 37.9 (8) years and 23.2 (3.3) kg/m^2^, respectively, and 54% percent were male. The overall prevalence of the metabolically unhealthy phenotype was 45%, and its prevalence increased with increasing BMI category as follows: 14.4% in BMI <18.5 kg/m^2^; 30% in 18.5 to 22.9 kg/m^2^; 52.9% in BMI of 23 to 24.9 kg/m^2^; 70.3% in BMI of 25 to 29.9 kg/m^2^; and 87.6% in BMI ≥30 kg/m^2^. Participants in higher BMI categories were more likely to be male and HEPA; to drink alcohol; to have higher levels of BP, glucose, uric acid, total cholesterol, LDL-C, triglycerides, ALT, hs-CRP, and HOMA-IR; and to have lower levels of HDL-C.

During 638,240.4 person-years of follow-up, 14,672 cases of incident cellulitis including outpatient and inpatient cases were identified (incidence rate of 23 per 10^3^ person-years). The median follow-up period was 4.2 years (interquartile range, 2.5–5.1). After adjustment for age, sex, center, year of screening exam, smoking status, alcohol intake, physical activity, total energy intake, educational level, and Charlson comorbidity index, multivariable-adjusted hazard ratios (95% CI) for incident cellulitis comparing BMIs of <18.5, 23–24.9, 25–29.9, and ≥30 kg/m^2^ with a BMI of 18.5–22.9 kg/m^2^ as the reference were 1.07 (1–1.15), 1.07 (1.02–1.11), 1.09 (1.04–1.13), and 1.19 (1.08–1.3), respectively (Table 2, Model 1). For further adjustment for total cholesterol, HDL-C, triglycerides, glucose, systolic BP, HOMA-IR and hsCRP, these associations remained significant and were similarly observed in both metabolically healthy and unhealthy phenotypes (Table 2, Model 2). Multivariable-adjusted HRs (95% CI) for incident cellulitis comparing BMIs <18.5, 23–24.9, 25–29.9, and ≥30 kg/m^2^ with a BMI of 18.5–22.9 kg/m^2^ as the reference were 1.04 (0.96–1.13), 1.13 (1.06–1.2), 1.10 (1.02–1.19), and 1.31 (1.01–1.71), respectively, in metabolically healthy individuals, whereas corresponding HRs (95% CI) were 1.08 (0.9–1.3), 1.05 (0.98–1.12), 1.13 (1.05–1.2), and 1.26 (1.12–1.42), respectively, in metabolically unhealthy individuals.

Table 3 shows the association between BMI categories and the development of cellulitis-related hospitalization in overall, metabolically healthy, and unhealthy individuals. During 670,455.4 person-years of follow-up, a total of 225 cases of new-onset cellulitis-related hospitalization were identified (incidence rate of 33.6 per 10^5^ person-years). The age- and sex-adjusted hazard ratios (95% CI) for cellulitis-related hospitalization comparing BMIs of <18.5, 23–24.9, 25–29.9, and ≥30 kg/m^2^ with a BMI of 18.5–22.9 kg/m^2^ as the reference were 0.54 (0.17–1.73), 1.59 (1.07–2.35), 2.58 (1.8–3.68), and 5.22 (3.11–8.73), respectively. These associations were observed in both metabolically healthy and unhealthy phenotypes with no significant interaction by metabolic health status (*p* for interaction = 0.073). After further adjustment for potential confounders (Table 3, Model 1), multivariable-adjusted HRs (95% CI) for cellulitis-related hospitalization comparing BMIs <18.5, 23–24.9, and 25–29.9 kg/m^2^ with a BMI of 18.5–22.9 kg/m^2^ as the reference were 0.82 (0.25–2.74), 2.01 (1.12–3.61), and 4.31 (2.51–7.42), respectively, in metabolically healthy individuals, whereas corresponding HRs (95% CI) comparing BMIs 23–24.9, 25–29.9, and ≥30 kg/m^2^ with a BMI of 18.5–22.9 kg/m^2^ as the reference in metabolically unhealthy individuals were 1.12 (0.66–1.9), 1.55 (0.97–2.48), and 3.86 (2.15–6.92), respectively. After further adjustment for LDL-C, HDL-C, triglycerides, glucose, SBP, hs-CRP, and HOMA-IR, the associations between BMI categories and cellulitis-related hospitalization were attenuated but remained significant (Table 3, Model 2).

We compared characteristics of participants included in the analysis and those who were excluded from the analysis due to missing data. Participants with missing data were more likely to be older, current smokers, alcohol drinkers, have fatty liver, and have higher levels of metabolic parameters and HOMA-IR than those included in the analysis (Supplementary Table S1). However, there were very small differences in all variables, except age, between the two groups.

## 4. Discussion

In this large-scale, prospective cohort study of 171,322 apparently healthy young and middle-aged men and women, we demonstrated the effect of obesity on cellulitis incidence according to metabolic health status. Increased BMI categories were associated with an increased risk of cellulitis in both metabolically healthy and unhealthy phenotypes. These associations were still observed even after adjustment of confounders and metabolic parameters in a dose–response manner. In particular, the association with cellulitis-related hospitalization was more pronounced than the association with cellulitis.

Previous data suggest that obese people are more likely than normal weight people to develop various infections. On the other hand, there is very limited data on the association between obesity and cellulitis, especially in the general population. In a recent Danish cohort study among 37,808 blood donors (considered healthy individuals), Kasperson et al. found that obesity based on self-reported (as opposed to measured) weight and height was associated with greater risk of skin and subcutaneous tissue infection only in men [14]. Another Danish cohort study of 75,001 Danish women showed that overweight and obesity using prepregancy BMI based on self-reported weight and height were associated with increased risk of cellulitis [27]. Furthermore, none of these studies have evaluated the effect of obesity on the cellulitis risk while considering coexisting metabolic abnormalities. This is, to our knowledge, the largest study to prove the association of obesity and cellulitis on a large cohort of healthy individuals according to metabolic health. In the current study of apparently healthy adults free of diabetes, increased BMI categories using measured anthropometry were associated with an increased risk of cellulitis as well as cellulitis-related hospitalization. These associations were observed in both metabolically healthy and unhealthy individuals even after further adjustment for metabolic parameters including hsCRP and HOMA-IR. Our study suggests that excessive adiposity, per se, can increase the risk of cellulitis independent of coexisting metabolic conditions.

In a detailed analysis of our study, obesity was more strongly associated with cellulitis-related hospitalization than cellulitis, including outpatient cases, in a dose–response manner. This association was prominent even in metabolically healthy individuals. Cellulitis often requires hospitalization for intravenous antibiotics, especially for patient who frequently have comorbid conditions or patients with severe manifestations. There is also a possibility that many obese patients were suboptimally dosed with oral antibiotics and then considered to have failed treatment, for which they were admitted to hospital for intravenous antibiotics. Unfortunately, information on the severity of cellulitis was not available in this study since HIRA data do not include disease severity [9] Thus, obesity, per se, appears to adversely affect the risk of cellulitis occurrence as well as the cellulitis requiring hospitalization. In metabolically healthy individuals, no cellulitis-related hospitalization was observed in the highest category of BMI ≥30 kg/m^2^ even though increased BMI categories were positively associated with increased risk of cellulitis-related hospitalization. A relatively small proportion of obese individuals with BMI ≥30 kg/m^2^ was metabolically healthy, and analysis was limited by the insufficient sample size of this group. Further studies with large sample sizes and longer follow-up are needed to confirm these findings.

The biological mechanism of the increased risk of infection among those with high BMI remains poorly understood. Obesity has a clear but not yet precisely defined effect on the immune response through a variety of immune mediators, which leads to susceptibility to infections [8]. The adipose tissue is now recognized an active organ involved in inflammation and immunity, producing and releasing a variety of pro-inflammatory and anti-inflammatory factors [8]. Adiponectin, one of the adipokines, is potently immunosuppressive [28], while leptin seems to be a protective component of the immune response [29]. Additionally, obese patients tend to have dry skin and impaired skin barrier repair, and their lymphatic flow might be impaired following an inflammatory state [30].

We note that our study has some limitations. First, we used BMI to classify obesity. BMI cannot distinguish fat tissue from lean mass. However, BMI has correlated well with fat mass and is widely used as an indicator of general fatness [31]. Second, smoking and alcohol use were assessed via a self-administered, structured questionnaire used in health checkup programs in Korea as part of the National Health Insurance plan [17]. Third, Health Insurance and Review Assessment (HIRA) in South Korea is designed and used in the process of reimbursing healthcare providers [32]. Discrepancies may occur between diagnoses entered in the data and diseases that a patient has in reality, and some degree of outcome misclassification is inevitable [32]. However, diagnosis in HIRA inpatient claims tends to be more accurate for severe conditions than acute and minor conditions and for inpatients than for outpatients [32]. We found a positive association between BMI category and cellulitis in both outpatient and inpatient settings, and this association was stronger in the case of hospitalization for cellulitis than in the case of cellulitis that was diagnosed at either outpatient or inpatient settings. Thus, the stronger association between BMI category and cellulitis hospitalization can be related to less misclassification errors of outcome rather than cellulitis diagnosed in an outpatient setting. Finally, our results derived from a sample of relatively healthy, young and middle-aged, educated Koreans with great access to health care resources, and they might not be generalizable to other ages and ethnic populations.

## 5. Conclusions

In conclusion, we found that increased BMI was independently associated with an increased risk of cellulitis and cellulitis-related hospitalization in both metabolically healthy and unhealthy individuals, indicating excessive adiposity, per se, is an independent risk factor for cellulitis. Better understating of the interplay between obesity and cellulitis has critical implications for community health, given that the prevalence of obesity has been on the rise steadily over the past decades, and the burden of cellulitis is high in each country [33,34]. Maintaining a normal healthy weight may help reduce the risk of future various infectious diseases as well as cellulitis.

## Figures and Tables

**Table 1:** Baseline characteristics of study participants by body mass index category.

Characteristic	Overall	BMI Category (kg/m^2^)					*p* for Trend
		<18.5	18.5–22.9	23–24.9	25–29.9	≥30	
Number	171,322	9982	77,595	38,177	40,499	5069	
Age (years) ^1^	37.9 (8)	34.5 (6.5)	37.2 (7.7)	39.1 (8.4)	39 (8.2)	36.7 (7.4)	<0.001
Male (%)	54	12.1	36.5	70.7	79.6	75	<0.001
Fatty liver (%)	25.9	0.5	7.4	29.5	57	84.3	<0.001
Current smoker (%)	22.5	7.9	15.6	27.0	32.8	34.5	<0.001
Alcohol intake (%) ^2^	23.2	8.1	15.9	27.8	34.5	35.7	<0.001
HEPA (%)	16.3	10.3	15.4	17.9	17.8	16.8	<0.001
Higher education (%) ^3^	79.7	79	79.5	80.5	80.2	75.7	0.675
Systolic BP (mmHg) ^1^	109.1 (12.9)	99.5 (9.8)	104.7 (11.5)	111.5 (11.7)	116 (11.9)	121.9 (12.9)	<0.001
Diastolic BP (mmHg) ^1^	69.8 (9.8)	64.4 (7.8)	66.9 (8.8)	71.2 (9.4)	74.2 (9.8)	77.1 (10.5)	<0.001
Glucose (mg/dL) ^1^	93.3 (8.4)	89.1 (7.5)	91.4 (7.8)	94.4 (8.1)	96.2 (8.5)	98 (9)	<0.001
Total cholesterol (mg/dL) ^1^	193.2 (33.9)	178 (28.7)	186.4 (31.6)	197.6 (33.7)	203.9 (34.8)	207.3 (35.6)	<0.001
LDL-C (mg/dL) ^1^	119.6 (31.8)	98.5 (24.5)	111.3 (29.1)	125.9 (30.9)	132.7 (31.5)	136.4 (31.6)	<0.001
HDL-C (mg/dL) ^1^	58.9 (15.1)	70.7 (14.3)	63.8 (14.7)	55.7 (13.3)	51 (12.1)	47.3 (10.9)	<0.001
Triglycerides (mg/dL) ^4^	89 (63–131)	62 (50–79)	73 (56–101)	100 (72–142)	126 (89–179)	149 (106–210)	<0.001
ALT (U/L) ^4^	18 (12–27)	12 (10–16)	14 (11–20)	20 (14–28)	26 (18–39)	37 (25–60)	<0.001
hsCRP (mg/L) ^4^	0.4 (0.2–0.9)	0.2 (0.2–0.4)	0.3 (0.2–0.6)	0.5 (0.3–0.9)	0.7 (0.4–1.3)	1.3 (0.7–2.6)	<0.001
HOMA-IR ^4^	1.16 (0.77–1.72)	0.80 (0.53–1.16)	0.96 (0.65–1.37)	1.21 (0.84–1.71)	1.62 (1.13–2.28)	2.67 (1.88–3.78)	<0.001
Total energy intake (kcal/d) ^2,5^	1491.2 (1098.9–1899.6)	1362.0 (991.0–1747.1)	1435.7 (1049.0–1830.5)	1527.6 (1138.9–1934.9)	1584.3 (1192.6–2011.2)	1652.9 (1236.3–2154.0)	<0.001
Charlson comorbidity index							
1–2 (%)	0.14	0.11	0.12	0.13	0.17	0.18	0.018
≥3 (%)	0.01	0	0.01	0.01	0	0.02	0.756

Data are ^1^ mean (standard deviation), ^4^ median (interquartile range), or percentage. ^2^ ≥20 g of ethanol per day; ^3^ ≥College graduate. ALT = alanine aminotransferase, BMI = body mass index, BP = blood pressure, HDL-C = high-density lipoprotein cholesterol, HEPA = health-enhancing physical activity, hsCRP = high-sensitivity C-reactive protein, HOMA-IR = homeostasis model assessment of insulin resistance, LDL-C = low-density lipoprotein cholesterol. ^5^ Among 141,911 participants with plausible estimated energy intake levels (within three standard deviations from the log-transformed mean energy intake).

**Table 2:** Development of cellulitis including outpatient and inpatient by body mass index category in metabolically healthy and unhealthy phenotypes.

BMI Category (kg/m^2^)	Person-Years	Incident Cases	Incidence Rate (Cases per 1000 person-years)	Age- and Sex-Adjusted HR (95% CI)	Multivariable-Adjusted HR ^1^ (95% CI)	
					Model 1	Model 2
Total (*n* = 171,322)						
<18.5	36,448.7	834	22.9	1.07 (0.99–1.15)	1.07 (1–1.15)	1.06 (0.98–1.14)
18.5–22.9	289,945.1	6315	21.8	1 (reference)	1 (reference)	1 (reference)
23–24.9	144,254.6	3415	23.7	1.07 (1.03–1.12)	1.07 (1.02–1.11)	1.08 (1.04–1.13)
25–29.9	150,300.0	3648	24.3	1.1 (1.05–1.15)	1.09 (1.04–1.13)	1.12 (1.07–1.18)
≥30	17,292.1	460	26.6	1.22 (1.11–1.34)	1.19 (1.08–1.31)	1.28 (1.15–1.42)
*p* for trend				<0.001	<0.001	<0.001
Metabolically healthy phenotype (*n* = 93,520)						
<18.5	30,930.5	708	22.9	1.05 (0.97–1.14)	1.06 (0.98–1.15)	1.04 (0.96–1.13)
18.5–22.9	198,867.9	4352	21.9	1 (reference)	1 (reference)	1 (reference)
23–24.9	65,448.3	1609	24.6	1.12 (1.05–1.19)	1.11 (1.04–1.17)	1.13 (1.06–1.20)
25–29.9	43,176.6	1022	23.7	1.08 (1–1.16)	1.06 (0.99–1.14)	1.1 (1.02–1.19)
≥30	2109.4	58	27.5	1.26 (0.97–1.63)	1.23 (0.95–1.60)	1.31 (1.01–1.71)
*p* for trend				0.013	0.0573	0.005
Metabolically unhealthy phenotype (*n* = 77,802)						
<18.5	5518.2	126	22.8	1.09 (0.91–1.30)	1.1 (0.92–1.32)	1.08 (0.9–1.3)
18.5–22.9	91,077.2	1963	21.6	1 (reference)	1 (reference)	1 (reference)
23–24.9	78,806.4	1806	22.9	1.05 (0.98–1.12)	1.04 (0.97–1.11)	1.05 (0.98–1.12)
25–29.9	107,123.4	2626	24.5	1.12 (1.05–1.19)	1.10 (1.04–1.17)	1.13 (1.05–1.2)
≥30	15,182.7	402	26.5	1.23 (1.1–1.37)	1.20 (1.08–1.34)	1.26 (1.12–1.42)
*p* for trend				<0.001	<0.001	<0.001

*p* = 0.37 for the overall interaction between metabolic health status and BMI category for incident cellulitis (adjusted model 1^1^). ^1^ Estimated from Cox proportional hazard models. Multivariable model 1 was adjusted for age, sex, center, year of screening exam, smoking status, alcohol intake, physical activity, total energy intake, educational level, and Charlson comorbidity index; model 2: model 1 plus adjustment for total cholesterol, HDL, triglycerides, glucose, hsCRP (high-sensitivity C-reactive protein), systolic blood pressure, and HOMA-IR (homeostasis model assessment of insulin resistance). CI = confidence interval, and HR = hazard ratio.

**Table 3:** Development of cellulitis admission by body mass index category in metabolically healthy and unhealthy phenotypes.

BMI Category (kg/m^2^)	Person-Years	Incident Cases	Incidence Rate (cases per 100,000 person-years)	Age- and Sex-Adjusted HR (95% CI)	Multivariable-Adjusted HR ^1^ (95% CI)	
					Model 1	Model 2
Total (*n* = 171,322)						
<18.5	38,246.8	3	7.8	0.54 (0.17–1.73)	0.55 (0.17–1.75)	0.57 (0.18–1.83)
18.5–22.9	303,902.3	54	17.8	1.00 (reference)	1 (reference)	1 (reference)
23–24.9	151,692.7	53	34.9	1.59 (1.07–2.35)	1.55 (1.05–2.3)	1.48 (0.99–2.21)
25–29.9	158,308.2	94	59.4	2.58 (1.8–3.68)	2.47 (1.73–3.53)	2.21 (1.5–3.26)
≥30	18,305.5	21	114.7	5.22 (3.11–8.73)	4.80 (2.86–8.05)	3.78 (2.1–6.81)
*p* for trend				<0.001	<0.001	<0.001
Metabolically healthy phenotype (*n* = 93,520)						
<18.5	32,421.6	3	9.3	0.83 (0.25–2.77)	0.82 (0.25–2.74)	0.78 (0.23–2.6)
18.5–22.9	208,329.0	27	13	1 (reference)	1 (reference)	1 (reference)
23–24.9	68,842.8	22	32	2.01 (1.12–3.61)	2.01 (1.12–3.61)	2.16 (1.19–3.93)
25–29.9	45,368.5	33	72.7	4.38 (2.54–7.54)	4.31 (2.51–7.42)	4.96 (2.74–8.99)
≥30	2233.1	0	–	–	–	–
*p* for trend				<0.001	<0.001	<0.001
Metabolically unhealthy phenotype (*n* = 77,802)						
<18.5	5825.2	0	–	–	–	–
18.5–22.9	95,573.3	27	28.3	1 (reference)	1 (reference)	1 (reference)
23–24.9	82,849.9	31	37.4	1.15 (0.68–1.94)	1.12 (0.66–1.9)	1.04 (0.61–1.77)
25–29.9	112,939.7	61	54	1.61 (1.01–2.58)	1.55 (0.97–2.48)	1.31 (0.8–2.14)
≥30	16,072.4	21	130.7	4.12 (2.31–7.36)	3.86 (2.15–6.92)	2.77 (1.45–5.29)
*p* for trend				<0.001	<0.001	0.006

*p* = 0.073 for the overall interaction between metabolic health status and BMI category for incident cellulitis-related admission (adjusted model 1^1^). ^1^ Estimated from Cox proportional hazard models. Multivariable model 1 was adjusted for age, sex, center, year of screening exam, smoking status, alcohol intake, physical activity, total energy intake, educational level, and Charlson comorbidity index; model 2: model 1 plus adjustment for total cholesterol, HDL, triglycerides, glucose, hsCRP (high-sensitivity C-reactive protein), systolic blood pressure, and HOMA-IR (homeostasis model assessment of insulin resistance). CI = confidence interval, and HR = hazard ratio.

## Supplementary Materials

The following are available online at https://www.mdpi.com/2077-0383/8/7/953/s1, Table S1: Baseline characteristics of study participants by body mass index category.
