# Supplementary material for: Metabolic Obesity Phenotypes and Risk of Cellulitis: A Cohort Study
Source: J Clin Med. 2019 Jun 30;8(7):953. doi: 10.3390/jcm8070953 (PMC6679047; doi:10.3390/jcm8070953)
Supplement: Supplementary file 1 [file jcm-08-00953-s001.pdf]

Supplemental Table S1. Baseline characteristics of study participants by body mass index category.

Characteristic	Overall	Missing Data Group	Final Inclusion Group	*p* Value
Number	210,038	38,716	171,322	
Age (years) [1]	38.8 (8.7)	43.1 (10.1)	37.9 (8)	<0.001
Male (%)	54.2	54.7	54	0.014
Fatty liver (%)	26.5	28.9	25.9	<0.001
Current smoker (%)	22.8	24.1	22.5	<0.001
Alcohol intake (%) [2]	24	27.4	23.2	<0.001
HEPA (%)	16.6	17.9	16.3	<0.001
Higher education (%) [3]	79.4	77.9	79.7	<0.001
Systolic BP (mmHg) [1]	109.2 (13)	109.8 (13.3)	109.1 (12.9)	<0.001
Diastolic BP (mmHg) [1]	69.9 (9.9)	70.6 (10)	69.8 (9.8)	<0.001
Glucose (mg/dL) [1]	93.5 (8.5)	94.4 (8.6)	93.3 (8.4)	<0.001
Total cholesterol (mg/dL) [1]	193.7 (34.0)	196.3 (34.1)	193.2 (33.9)	<0.001
LDL–C (mg/dL) [1]	120.2 (31.9)	122.9 (32.2)	119.6 (31.8)	<0.001
HDL–C (mg/dL) [1]	58.9 (15.2)	59.1 (15.5)	58.9 (15.1)	0.013
Triglycerides (mg/dL) [4]	89 (63–132)	92 (65–135)	89 (63–131)	<0.001
ALT (U/L) [4]	18 (13–27)	18 (13–26)	18 (12–27)	0.039
hsCRP (mg/L) [4]	0.4 (0.2–0.9)	0.5 (0.3–1.1)	0.4 (0.2–0.9)	<0.001
HOMA–IR [4]	1.17 (0.77–1.73)	1.22 (0.81–1.8)	1.16 (0.77–1.72)	<0.001
Total energy intake (kcal/d) [2,5]	1482.5 (1089–1889.6)	1439.6 (1038.8–1842.2)	1491.2 (1098.9–1899.6)	<0.001
Charlson comorbidity index				
1–2 (%)	0.15	0.19	0.14	0.014
≥3 (%)	0.01	0.01	0.01	0.773

Data are [1] mean (standard deviation), [4] median (interquartile range), or percentage. [2] ≥20 g of ethanol per day; [3] ≥College graduate. ALT = alanine aminotransferase, BMI = body mass index, BP = blood pressure, HDL-C = high-density lipoprotein cholesterol, HEPA = health-enhancing physical activity, hsCRP = high sensitivity C-reactive protein, HOMA-IR = homeostasis model assessment of insulin resistance, LDL-C = low-density lipoprotein cholesterol. [5] among 171,582 participants with plausible estimated energy intake levels (within three standard deviations from the log-transformed mean energy intake).
